# 肿瘤突变负荷应用于肺癌免疫治疗的专家共识

**DOI:** 10.3779/j.issn.1009-3419.2021.101.40

**Published:** 2021-11-20

**Authors:** 中国临床肿瘤学会血管靶向治疗专家委员会, 中国临床肿瘤学会非小细胞肺癌专家委员会

**Keywords:** 肺肿瘤, 免疫治疗, 免疫检查点抑制剂, 肿瘤突变负荷, 专家共识, Lung neoplasms, Immunotherapy, Immune checkpoint inhibitors, Tumor mutational burden, Expert consensus

## Abstract

肺癌是全球范围内发病率和死亡率最高的恶性肿瘤之一。免疫检查点抑制剂（immune checkpoint inhibitors, ICIs），包括程序性死亡受体1（programmed cell death 1, PD-1）抗体、程序性死亡受体1配体（programmed cell death ligand 1, PD-L1）抗体和细胞毒性T淋巴细胞相关蛋白4（cytotoxic T lymphocyte antigen 4, CTLA-4）抗体，给部分晚期肺癌患者带来了显著的生存获益，改变了晚期肺癌的治疗格局。既往研究表明，PD-1/PD-L1抗体在晚期非小细胞肺癌（non-small cell lung cancer, NSCLC）中的客观缓解率只有20%左右。所以临床亟需可靠的生物标志物协助筛选ICIs潜在获益人群，提高治疗响应率。肿瘤突变负荷（tumor mutational burden, TMB）是除PD-L1表达以外新兴的免疫治疗标志物。肺癌中PD-L1表达与TMB的相关性不大，评估TMB可扩大免疫治疗的获益人群。然而在临床实践中，TMB的检测、阈值的确定和临床指导策略仍然没有形成规范。本共识将对TMB检测和应用场景给出指导性建议，以促进TMB在肺癌免疫治疗中应用的规范化。

## Abstract (en)

Lung cancer is one of the malignant tumors with the highest morbidity and mortality in the world. Immune checkpoint inhibitors (ICIs), including programmed cell death 1 (PD-1) antibody, programmed cell death ligand 1 (PD-L1) antibody, and cytotoxic T lymphocyte associated protein 4 (CTLA-4) antibody. It has brought significant survival benefits to some patients with advanced lung cancer and changed the treatment pattern of advanced lung cancer. Previous studies have shown that the objective response rate of PD-1/PD-L1 antibody in advanced non-small cell lung cancer (NSCLC) is only about 20%. So reliable biomarkers are urgently needed to screen out the potential benefit population of ICIs and improve the clinical response rate. Tumor mutational burden (TMB) is an emerging biomarker of immunotherapy in addition to PD-L1 expression. There is little correlation between PD-L1 expression and TMB in lung cancer. It is estimated that TMB can expand the benefit population of immunotherapy. However, in clinical practice, the detection of TMB, the determination of cut-off value and the clinical guidance strategy are still not standardized. This consensus will give guiding suggestions on the detection and application scenarios of TMB, so as to promote the standardization of TMB application for immunotherapy in lung cancer.

无论是非小细胞肺癌（non-small cell lung cancer, NSCLC）还是小细胞肺癌（small cell lung cancer, SCLC），均有部分患者可从免疫检查点抑制剂（immune checkpoint inhibitors, ICIs）治疗中获益。目前，预测程序性细胞死亡受体1（programmed cell death 1, PD-1）及其配体（programmed cell death ligand 1, PD-L1）抗体疗效的生物标志物主要有PD-L1表达、微卫星不稳定性和肿瘤突变负荷（tumor mutational burden, TMB）。TMB概念起源于2013年*Nature*发表的一项研究^[1]^。在30个癌种7, 000多个标本中，研究者通过全基因组测序（whole genome sequencing, WGS）和全外显子测序（whole exome sequencing, WES）技术分析了突变图谱，描述了不同癌种样本中每百万碱基（megabase, Mb）的突变数量。2014年的一项黑色素瘤研究^[2]^发现，免疫治疗的响应率与肿瘤突变数目有一定的相关性，通过WES检出错义突变数量大于100的患者接受免疫治疗后具有更长的总生存期（overall survival, OS），这是首个验证TMB和免疫治疗疗效相关性的研究。2015年，首个TMB与NSCLC免疫治疗疗效的研究^[3]^发表于*Science*，该研究发现高于中位TMB的NSCLC患者具有更长的无进展生存期（progression-free survival, PFS）。此后，CheckMate-026、CheckMate-227等多项大型研究证实了TMB对NSCLC免疫治疗疗效的预测作用。2017年，*Genome Medicine*发表的一项10万例实体瘤患者研究^[4]^，探索了靶向捕获测序panel与WES检测TMB的相关性，证实了panel检测TMB的可行性与可靠性。2019年中国临床肿瘤学会（Chinese Society of Clinical Oncology, CSCO）指南和美国国家综合癌症网络（National Comprehensive Cancer Network, NCCN）指南均将TMB纳入晚期肺癌的分子病理检测范围。2020年，美国食品药品监督管理局（Food and Drug Administration, FDA）批准Pembrolizumab单药用于治疗高TMB且既往接受治疗后病情进展的不可手术或转移性实体瘤患者。Pembrolizumab成为全球首个以TMB作为标志物而获批的抗肿瘤药物。但临床实践中TMB的检测和评估缺乏统一的标准，这极大限制了其临床应用。虽然《肿瘤突变负荷检测及临床应用中国专家共识（2020年版）》已发布^[5]^，但TMB在肺癌免疫治疗临床应用中的相关规范仍有待统一。为促进TMB在肺癌免疫治疗中应用的规范化，协作组组织国内肺癌领域权威专家，综合国内外高质量文献，形成《肿瘤突变负荷应用于肺癌免疫治疗的专家共识》，对TMB的定义、临床意义和临床应用给出指导性建议。

## 1 TMB的定义

TMB是指肿瘤基因组内存在的体细胞突变位点数量，可以间接反映肿瘤产生新生抗原的能力^[6]^。由于早期研究多基于WES检测^[7-10]^，因此TMB通常是指单位基因组外显子编码区域（外显子组，exome）的突变数量（mutations, muts），单位为muts/exome。虽然WES是检测TMB的金标准^[11]^，但WES时间成本和分析成本较高。经过多项大样本研究验证后，TMB检测从WES扩展到了更切合临床实际的靶向二代测序（next-generation sequencing panel, NGS panel）^[12-17]^。靶向测序的基因检测位点比外显子组少，由于不同平台检测方法和测序覆盖的外显子区域长度不同，TMB也被定义为肿瘤基因组区域中每兆碱基（megabase, Mb）发生的碱基替换突变和插入缺失突变的数量总和，单位为muts/Mb。

不同研究纳入TMB计算的突变类型不同。例如，CheckMate-026研究^[9]^中，TMB被定义为通过WES测序肿瘤组织样本中体细胞非同义突变数量的总和。在NGS panel检测TMB的研究中，纳入TMB计算的是体细胞编码区中碱基替换突变和插入缺失突变，部分NGS panel计算TMB也同时纳入了同义突变，而胚系变异、核苷酸多态性位点、明确的抑癌基因及驱动基因热点突变则不计算在内^[4, 18, 19]^。

## 2 TMB的检测方法

### 2.1 WES

WES是测定肿瘤TMB的金标准。NSCLC、黑色素瘤和尿路上皮癌等癌种的早期免疫治疗研究均使用WES计算TMB^[20-22]^。由于同义突变产生新生抗原的可能性较低，利用WES计算TMB时仅纳入非同义突变。但WES检测成本较高、对样本要求较高、数据分析较为复杂，在临床应用中有较大的局限性。到目前为止，FDA仅批准了一款采用WES技术检测TMB的产品（Omics Core^SM^, NantHealth）。

### 2.2 NGS panel

通过NGS panel检测TMB的计算标准为每百万碱基中单碱基突变（可以纳入同义突变）以及短插入和缺失突变。NGS panel检测TMB需要与WES检测TMB有较高的一致性。有研究^[11]^指出，对于突变频率高的肿瘤，较小的NGS panel足够用于TMB评估。但也有一些证据^[23, 24]^发现检测的基因数越多，TMB的检测结果与WES的一致性越高，NGS大panel可能更适合评估TMB。对于TMB较高的样本，不同大小的NGS panel检测结果差异可能不显著；而对于TMB较低的样本，检测结果的不一致性显著增加。目前一般认为NGS panel ≥0.8 Mb可以较好地评估肿瘤组织TMB水平^[11]^。测序深度是每个碱基读长的平均次数。WES的测序深度约100×，对于等位基因突变频率大于15%的位点检测较灵敏^[25]^。NGS panel的测序深度应大于500×，可提高低频突变的检测敏感性^[26, 27]^。目前已有两款基于组织的panel产品（FoundationOne CDx，Foundation Medicine和PGDx Elio Tissue Complete，Personal Genome Diagnostics）和一款基于血液的panel产品（FoundationOne Liquid CDx, Foundation Medicine）获得FDA批准用于TMB检测。计算机模拟及临床实践均证明经这些NGS panel检测的TMB与经WES检测的TMB有较好的一致性，并且在临床试验中证实可以预测免疫治疗疗效。此外，测序操作建议选用国家药品监督管理局（National Medical Products Administration, NMPA）或者FDA批准的测序仪器。通过NGS panel检测TMB需经过模型建立、虚拟验证、技术验证和临床验证。

## 3 影响TMB检测的因素

样本收集阶段、DNA处理阶段、测序阶段、生物信息分析阶段和报告生成阶段均会影响TMB检测的可靠性。样本收集阶段主要包括样本类型、肿瘤类型、肿瘤异质性和克隆进化等影响因素；DNA处理阶段包括DNA质量和数量、文库构建等影响因素；测序阶段包括DNA捕获区域、测序深度、覆盖读长、测序平台等影响因素；生物信息分析阶段包括突变类型、胚系突变过滤、等位基因突变频率等影响因素^[28-30]^；报告生成阶段包括瘤种分类、患者人群、患者数量、TMB排序标准等影响因素^[31, 32]^。除了考虑技术因素，流程监管、样本收集和处理质控以及样本运送时长等因素可能也会影响样本质量，从而影响检测结果^[6]^。

## 4 TMB的临床意义

免疫检查点分子可负向调控细胞毒性T细胞对肿瘤细胞的杀伤活性，从而抑制新生抗原驱动的抗肿瘤免疫反应^[33]^。PD-1抗体、PD-L1抗体和细胞毒性T淋巴细胞相关蛋白4（cytotoxic T lymphocyte antigen 4, CTLA-4）抗体能够与免疫检查点分子结合，恢复细胞毒性T细胞对肿瘤细胞杀伤活性，启动抗肿瘤免疫反应。高TMB的肿瘤往往携带较高水平的新生抗原，肿瘤特异性体细胞突变所产生的新生蛋白或其降解产物被主要组织相容性复合体递呈到肿瘤细胞表面，形成肿瘤新生抗原，可能导致T细胞的激活，故能提高ICIs的治疗敏感性。现有证据表明，TMB是晚期肺癌免疫治疗的疗效预测因子和预后的影响因素。

从2015年至今，已经有大量临床研究探索了TMB对肺癌免疫治疗疗效的预测作用。KEYNOTE-158是其中一项里程碑式研究^[34]^，该研究纳入10个癌种共计1, 032例难治性实体瘤患者，结果表明Pembrolizumab在高TMB患者中总体客观缓解率（objective response rate, ORR）为29%，而低TMB患者ORR仅为6%。基于该结果，Pembrolizumab被FDA批准用于高TMB且既往治疗后疾病进展的不可手术或转移性实体瘤患者，其中高TMB定义为组织TMB≥10 muts/Mb。除了基于肿瘤组织检测TMB，通过液体活检技术评估血液TMB（blood-based TMB, bTMB）水平并预测免疫治疗疗效也展开了初步探索。2018年发表于*Nature Medicine*的一项回顾性研究^[35]^分析了bTMB与Atezolizumab疗效的相关性，该研究发现高bTMB的晚期NSCLC患者接受Atezolizumab治疗后ORR和PFS得到显著改善。2019年发表于*JAMA Oncology*的另一项研究^[24]^证实了通过NGS panel计算的bTMB同样能够预测晚期NSCLC患者接受免疫治疗的ORR和PFS。2020年发表在*Journal of Thoracic Oncology*的一项研究^[23]^进一步优化了bTMB算法，证实LAF-bTMB（low allele frequency, bTMB）能够有效地预测晚期NSCLC患者接受免疫治疗的ORR、PFS以及OS。这些研究表明，bTMB在预测免疫治疗疗效中具有一定前景。

## 5 TMB应用于肺癌免疫治疗共识

**共识一：WES是TMB检测的金标准，但目前测序成本和分析难度较高。NGS panel与WES的TMB检测结果具有高度一致性，经过验证的NGS panel可作为临床检测TMB的替代方式。**

TMB检测的金标准是WES。FDA已批准了一款基于WES技术检测TMB的产品（Omics Core^SM^），其包含19, 396个基因，覆盖DNA全长39 Mb，其中编码区（coding sequence, CDS）长度约为33.7 Mb。WES测序成本高、分析难度大，因此NGS panel测序技术在临床上得到了广泛应用。需要指出的是，NGS panel检测的TMB应与WES检测的TMB具有高度一致性才能指导临床选用ICIs^[31]^。

通过NGS检测TMB需经过模型建立、虚拟验证、技术验证和临床验证。模型建立指：在设计NGS panel时，应建立理论计算模型，对纳入不同基因数的NGS panel和WES进行TMB相关性比对。基因数量确定后，基因选择需符合以下标准：①权威指南推荐和有高等级循证医学证据的基因；②有高质量研究支持的上述基因相关的上下游通路基因；③与免疫治疗相关的基因，包括可能的敏感基因、原发或继发耐药基因、与免疫超进展发生相关的基因；④与TMB相关的基因，例如DNA损伤修复（DNA damage repair, DDR）相关基因等^[36]^。纳入随机选择的基因和通过筛选的基因分别计算TMB，比对NGS panel和WES检测TMB的相关性。此外，纳入同义突变对于NGS panel与WES检测TMB相关性的影响也应考虑在内。虚拟验证指：通过公共数据库比对待验证NGS panel及已获批NGS panel计算TMB与WES计算TMB的相关性。通过公共队列数据比对NGS panel计算的TMB能否区分临床获益。技术验证是指利用WES和NGS panel同时检测临床肿瘤样本，评估两种方法所得TMB的相关性是否较高。临床验证指：通过前瞻性或回顾性临床研究，验证NGS panel检测所得的TMB是否可用于提示免疫治疗疗效。

经过验证的NGS panel可用于替代WES检测TMB。FDA批准用于检测TMB的NGS panel产品（表 1）均与WES测序进行了对比研究，两者检测所得TMB结果高度一致。另一款FDA批准的NGS panel产品MSK-IMPACT在获批的临床应用中未包含TMB，但MSK-IMPACT在既往临床研究中被证明可用于替代WES进行TMB检测，体现了其临床应用价值。

**表 1:** FDA批准的TMB检测panel参数

产品名	基因数	覆盖DNA长度	覆盖CDS长度
FoundationOne CDx	324	2.2 Mb	0.8 Mb
PGDx Elio Tissue Complete	507	2.23 Mb	1.33 Mb
FDA：美国食品药品监督管理局；TMB：肿瘤突变负荷；CDS：编码区			

**共识二：通过病理质控的石蜡包埋肿瘤组织方可用于TMB检测。在肿瘤组织获取困难或石蜡包埋样本中肿瘤细胞含量不足的情况下，晚期NSCLC患者可选择外周血进行基于ctDNA的bTMB检测。**

石蜡包埋样本是临床上评估TMB最常用的标本类型。经石蜡包埋的肿瘤组织用于TMB检测之前应经过病理质控。手术组织取材大小应在5 mm×5 mm×3 mm及以上，取材时尽量选取肿瘤组织多的区域，避免坏死组织。组织离体后尽快（10 min内）浸入足量的10%中性缓冲福尔马林固定液中固定。穿刺活检样本取材直径应不小于1 mm，长度不小于10 mm，且不少于1条穿刺样本。穿刺取材时尽量选取肿瘤组织多的区域，避开坏死组织，若含坏死组织较多，建议重新活检。石蜡切片厚度约4 μm-5 μm。检测TMB时，手术来源的石蜡切片至少需要5张，穿刺样本或小标本切片需10张以上。为保证TMB检测结果的可靠性，建议送检1年内的石蜡包埋样本。既往有研究发现6个月内的蜡块基因组信息保留相对稳定，因此送检6个月内的蜡块更佳^[37]^。在临床实践中，DNA含量过低可能会影响TMB的评估^[38-40]^。根据FDA获批的TMB检测产品说明书显示，送检样本需至少含有50 ng肿瘤DNA。影响石蜡包埋样本提取DNA的因素包括：样本可用性^[41-44]^、收集、准备和固定（对于中性福尔马林固定的手术标本最佳固定时间约24 h，对于穿刺活检标本固定时间约12 h^[45]^）、样本肿瘤细胞含量（FDA批准的TMB检测产品对肿瘤纯度要求均≥20%）。应充分考虑肿瘤组织来源异质性对TMB结果的影响。研究^[46]^发现原发灶TMB和远处器官转移灶TMB差异不明显，而转移淋巴结来源的TMB则显著低于原发灶TMB，因此原发灶和远处器官转移灶均可用于评估TMB。此外，全血样本或正常组织也应同时采集，用于过滤胚系突变。全血标本采集推荐使用游离核酸保存管或含乙二胺四乙酸盐的抗凝采血管，约8 mL-10 mL。正常组织石蜡切片与肿瘤组织切片数量相似。

在肿瘤组织获取困难或石蜡包埋样本中肿瘤细胞含量不足的情况下，外周血循环肿瘤DNA（circulating tumor DNA, ctDNA）也是可选择的一种样本类型。相比于组织，使用ctDNA检测bTMB具有创伤性小、可重复检测等优势。血液样本推荐使用游离核酸保存管采集，并在说明书规定保存时间范围内分离血浆。分离后的血浆应按照核酸提取试剂盒说明书尽快提取血浆游离DNA（cell free DNA, cfDNA），DNA浓度采用核酸定量试剂盒定量。如无法及时提取cfDNA，应将血浆置于-80 ℃冰箱保存，并避免反复冻融。目前，使用ctDNA评估bTMB的技术已通过了方法验证和临床验证^[47-49]^。大多数研究^[35, 50]^认为组织和ctDNA评估的TMB一致性较高。尽管ctDNA检测bTMB的敏感性较组织低^[48, 51, 52]^，基于ctDNA评估bTMB依然具有潜在的临床应用前景。除了ctDNA，胸腔积液、脑脊液等液体活检样本用于TMB评估和指导临床用药的证据尚不充分，本共识不做讨论。

对于晚期NSCLC，基于组织检测的高TMB患者比低TMB患者接受免疫治疗后具有更高的ORR、更长的中位PFS^[9, 30, 53]^。类似地，基于ctDNA检测的B-F1RST研究^[54]^中，Atezolizumab一线治疗高bTMB和低bTMB的NSCLC患者的ORR分别为36.4%和6.4%，中位PFS分别为9.5个月和2.8个月。POPLAR和OAK研究的回顾性分析^[24, 35]^发现，高bTMB的患者PFS获益大于低bTMB的患者，但OS在高bTMB和低bTMB患者中无明显差异。然而，改进bTMB算法后的一项研究^[23]^表明，低丰度的bTMB可预测免疫治疗患者的OS获益。

CheckMate-032研究结果提示，基于组织检测的高TMB SCLC患者接受Nivolumab联合Ipilimumab后ORR、中位PFS以及中位OS均优于中低TMB患者^[55, 56]^。一项SCLC回顾性研究^[57]^也发现，基于组织检测的高TMB患者比低TMB患者具有更长的中位PFS和中位OS。目前，基于外周血ctDNA检测的bTMB相关研究在SCLC中的报道尚少。

**共识三：既往未接受过免疫治疗的晚期NSCLC患者，在接受免疫单药治疗前推荐进行TMB检测。TMB预测SCLC一线免疫治疗疗效的临床证据尚不充分，暂不作为提示免疫一线治疗疗效标志物，但可考虑作为二线及后线免疫治疗的疗效标志物。**

晚期NSCLC相关研究^[9]^显示，TMB对免疫一线治疗、二线治疗和后线治疗均有一定预测作用。例如，CheckMate-026研究表明，高TMB的NSCLC患者接受Nivolumab一线治疗的ORR为47%，而接受化疗的ORR仅为28%，中位PFS分别为9.7个月和5.8个月（HR=0.62, 95%CI: 0.38-1.00）；而中、低TMB患者接受免疫治疗的中位PFS显著劣于化疗（4.1个月*vs* 6.9个月；HR=1.82，95%CI：1.30-2.55）。以二线治疗为主的免疫治疗研究发现，高TMB的NSCLC患者比低TMB的患者具有更高的ORR、更长的中位PFS和OS^[30, 53]^。在以后线治疗为主的KEYNOTE-001研究中，高TMB的NSCLC患者中位PFS为14.5个月，而低TMB患者中位PFS仅为3.7个月^[3, 58]^。MYSTIC研究结果^[59]^表明，高TMB患者和低TMB患者接受PD-L1抑制剂Durvalumab治疗的中位OS分别为18.6个月和10.1个月。OAK和POPLAR研究的回顾性分析结果^[35]^提示，高bTMB的NSCLC患者接受Atezolizumab治疗的ORR和中位PFS显著优于化疗。这些数据表明，对于既往没有接受过免疫治疗的晚期NSCLC患者，TMB检测可以指导其选用ICIs单药治疗。

对于SCLC，IMpower-133研究亚组分析并未显示bTMB与Atezolizumab联合化疗的疗效具有相关性^[55]^。一项SCLC二线及后线免疫治疗研究（CheckMate-032）^[56]^发现，相比于低、中TMB患者，高TMB SCLC患者接受Nivolumab单药治疗的中位PFS和OS并未显著延长，但接受Nivolumab联合Ipilimumab治疗的高TMB患者中位PFS和OS显著延长。KEYNOTE-158研究^[34]^纳入了76例后线SCLC患者，高TMB组Pembrolizumab治疗的ORR达到29.4%，而低TMB组为9.5%。此外，一项回顾性研究^[57]^发现接受二线及后线免疫治疗的高TMB SCLC患者具有显著更长的中位PFS和OS。由此可见，TMB并不能预测SCLC一线免疫联合化疗的疗效，但对后线免疫治疗疗效具有一定的预测价值。

**共识四：不推荐PD-1/PD-L1抗体联合化疗的晚期NSCLC或SCLC患者接受TMB检测。**

ICIs联合治疗方式包括ICIs联合化疗、双ICIs联合治疗、ICIs联合抗血管生成治疗、ICIs同时联合化疗及抗血管生成治疗等。Pembrolizumab联合化疗的KEYNOTE-021、KEYNOTE-189和KEYNOTE-407回顾性分析发现，高TMB不是预测Pembrolizumab联合化疗疗效的独立预测因素。从国内PD-1单抗药物的肺癌临床数据^[60]^可知，Sintilimab联合化疗（培美曲塞/顺铂）的Ib期研究中TMB与ORR无显著相关。对于接受Atezolizumab联合化疗的SCLC患者，bTMB也不是OS获益的预测因素^[55]^。因此，对于晚期NSCLC或SCLC患者，TMB并不能预测PD-1/PD-L1抗体联合化疗的疗效。

目前，双ICIs联合治疗晚期肺癌主要方式为PD-1/PD-L1抑制剂联合CTLA-4抑制剂。CheckMate-227研究中，当肿瘤细胞PD-L1≥1%、TMB≥10 muts/Mb时，接受Nivolumab联合Ipilimumab治疗的NSCLC患者比接受Nivolumab治疗患者的中位PFS更长（7.1个月*vs* 4.2个月）；TMB < 10 muts/Mb时，接受Nivolumab联合Ipilimumab治疗的患者仅有3.2个月的中位PFS^[12]^。MYSTIC研究中，bTMB≥20 muts/Mb时接受Durvalumab联合Tremelimumab治疗的NSCLC患者中位PFS及OS均长于Durvalumab单药治疗的患者^[59]^，而bTMB < 20 muts/Mb时结果相反。对于高TMB的SCLC患者，接受Nivolumab联合Ipilimumab治疗患者的中位PFS和中位OS分别为7.8个月和22个月，而接受Nivolumab治疗患者的中位PFS和中位OS仅有1.4个月和5.4个月。对于低TMB的SCLC患者，接受Nivolumab联合Ipilimumab治疗患者的中位PFS和中位OS分别为1.5个月和3.4个月，而接受Nivolumab治疗患者的中位PFS和中位OS分别为1.3个月和3.1个月^[56]^。由此可见，高TMB的NSCLC或SCLC患者接受Nivolumab联合Ipilimumab治疗获益更明显。需要特别强调的是，尽管高TMB可以预测PD-1/PD-L1抑制剂联合CTLA-4抑制剂的疗效，但CTLA-4抑制剂在我国目前尚未获批肺癌适应证。因此，对于PD-1/PD-L1抑制剂联合CTLA-4抑制剂治疗的晚期NSCLC或SCLC患者是否接受TMB检测暂不做推荐。

ICIs联合抗血管生成治疗、ICIs同时联合化疗和抗血管生成治疗中TMB的临床指导价值尚无充足的循证医学证据，有待于将来进一步探索。

**共识五：暂不推荐TMB用于预测免疫新辅助治疗疗效。**

相比于晚期NSCLC，TMB在早期NSCLC免疫新辅助治疗中的研究仍不多。2018年*New England Journal of Medicine*发表了一项Nivolumab用于I期-IIIa期NSCLC患者新辅助治疗的研究^[61]^，显著病理缓解（major pathological response, MPR）率为45%；达到MPR的患者和未达到MPR的患者具有的突变位点数量分别为311个和74个，具有显著统计学差异（*P*=0.01）。但在另一项免疫新辅助研究^[62]^中，Nivolumab联合Ipilimumab治疗的MPR为33%，MPR和TMB高低状态无显著相关性。由于这些均为小样本临床研究，未来还需要更多研究探索TMB在免疫新辅助治疗中的预测价值。

**共识六：目前尚没有公认的区分高、低TMB的cut-off值，推荐各免疫治疗药物根据各自临床研究数据确定cut-off值，并进行前瞻性验证。**

目前，区分高、低TMB的cut-off值并无统一界定，不同的研究采用不同的方法确定cut-off值。一种方式是通过训练集确定TMB cut-off值^[3, 63]^。例如，KEYNOTE-001研究的训练集TMB cut-off值为178 muts/exome，患者持续临床缓解的敏感性和特异性分别为100%和67%。TMB≥178 muts/exome的患者ORR为63%，而TMB < 178 muts/exome的患者ORR为0。对于中位PFS，TMB≥178 muts/exome的患者较TMB < 178 muts/exome的患者延长10.8个月。另一种方式是通过分位法确定TMB cut-off值。分位法目前有二分位（排序前50%为高TMB）、三分位（排序前30%为高TMB）、四分位（排序前25%为高TMB）、五分位（排序前20%为高TMB）和十分位（排序前10%为高TMB）。不同研究采用相同分位时，免疫治疗的获益有所不同^[53, 64]^。同一研究采用不同分位值时，免疫治疗的疗效是否随TMB升高而提高也具有争议。例如，发表于*Nature Genetics*的一项回顾性研究^[65]^发现免疫治疗获益随TMB的升高而提高。但在CheckMate-568研究中，TMB≥10 muts/Mb时ORR为43.8%，而TMB≥15 muts/Mb时ORR仅为39.3%^[63]^。在另一项研究^[53]^中，TMB排序前25%、50%、75%和90%患者的疾病控制率分别为24.1%、25.1%、25.7%和27.7%。这可能和纳入分析的患者数量、免疫治疗药物、NGS panel、TMB计算方法和研究设计类型等因素有关。建议临床尽可能采用循证医学证据较为充分的NGS panel进行TMB检测；并建议各类免疫治疗药物在开展临床研究时尽可能使用经过验证的NGS panel作为伴随诊断工具，根据各自临床研究数据确定TMB的cut-off值，并通过前瞻性研究加以验证。

**共识七：TMB检测报告应呈现纳入TMB计算的基因变异类型、TMB排序以及支持TMB排序的参考数据库，并提供辅助临床决策所需要的医学证据。**

WES检测TMB和NGS panel检测TMB所纳入的基因变异类型不同。对于WES检测TMB而言，仅纳入了肿瘤组织样本中体细胞非同义突变。而对于NGS panel，纳入TMB计算的则是体细胞编码区中碱基替换突变和插入缺失突变，有的研究还纳入了同义突变。无论是WES还是NGS panel检测TMB，胚系变异、核苷酸多态性位点、明确的抑癌基因及驱动基因热点突变均不计算在内。虽然纳入不同基因变异类型计算的TMB在肺癌ICIs临床研究中均有获益报道，但是对于TMB基因变异类型仍无统一规范。因此，TMB检测报告应呈现TMB计算纳入的变异类型，同时推荐报告中WES检测TMB的单位统一为muts/exome，NGS检测TMB的单位统一为muts/Mb。

如共识六所述，TMB cut-off值主要通过训练集或分位法确定。无论是哪种方法确定，高TMB患者在肺癌ICIs临床研究中均有获益报道。与计算TMB所纳入的基因变异类型相似，目前尚没有公认的TMB cut-off值。因此，TMB检测报告应呈现TMB排序以及支持该排序的参考数据库。此外，TMB检测报告应具备尽量全面的、更新及时的高级别循证医学证据用于辅助临床决策。医学证据包括TMB计算标准的相关文献、TMB cut-off值确定标准的相关文献、TMB技术验证和临床验证的相关文献、报告中TMB高低标准与免疫治疗疗效相关性的相关文献等。医学证据要求准确，应参考临床研究结论优先于临床前研究结论，前瞻性研究结论优先于回顾性研究结论的顺序进行展示和解读。

**共识八：建议联合PD-L1表达、免疫治疗相关基因、组织和循环中的免疫细胞等信息，对TMB指导肺癌免疫治疗进行综合解读。**

目前，PD-L1和NGS检测是临床上预判免疫治疗疗效应用最多的检测手段。PD-L1表达在NSCLC免疫治疗中的循证医学证据最为充分。TMB与PD-L1表达相关性不大^[30, 53]^。因此，临床实践中应将TMB检测结果与PD-L1表达结合起来进行解读。在一项晚期NSCLC免疫治疗研究^[66]^中，TMB高且PD-L1高表达的患者的ORR可达62.5%，而TMB高且PD-L1低表达的患者ORR为43.9%，TMB低且PD-L1高表达的患者ORR仅有27.3%。TMB高且PD-L1高表达的患者的中位PFS最长（HR=0.42, 95%CI: 0.30-0.58, *P* < 0.001）。在Rizvi等^[53]^的研究中也得到了相似的结论。除TMB数据以外，NGS还可以提供详细的肿瘤体细胞基因突变信息。一般认为，携带*EGFR*、*ALK*重排、*ROS1*融合、*STK11*/*LKB1*、*KEAP1*、*PTEN*、*JAK2*、*B2M*、*MDM2*/*MDM4*扩增等免疫耐药基因变异的肺癌患者，接受免疫单药疗效较差，甚至可能发生快速进展或超进展。而携带DDR通路基因突变的患者，由于DNA损伤修复基因功能受损，导致TMB增高，对免疫治疗可能更为敏感。王洁教授团队^[36]^前期研究发现，如果肿瘤细胞同时携带2条DDR通路基因突变，则其对免疫治疗疗效更佳。吴一龙教授团队^[67]^的研究结果表明，携带*TP53*和*KRAS*基因突变的肺腺癌患者PD-L1表达显著增高，免疫治疗疗效也优于野生型患者。因此，当TMB、PD-L1均为高表达且没有携带耐药基因突变时，强烈推荐使用免疫治疗；而当以上检测结果不一致时，需谨慎考虑使用免疫治疗，尤其是免疫单药治疗。

现有的研究数据^[10, 68-70]^表明，人类白细胞抗原（human leukocyte antigen, HLA）分型、肿瘤免疫微环境、肿瘤新生抗原和肠道菌群多样性等指标均与肺癌免疫治疗有一定相关性。例如，NSCLC患者肿瘤组织免疫微环境中PD-1^+^CD8^+^ T细胞、PD-L1^+^CD8^+^ T细胞、PD-L1^+^CD68^+^巨噬细胞水平与免疫治疗应答呈正相关^[71-73]^。此外，外周血CD8^+^ T细胞可能也与免疫治疗应答相关^[74]^。这些检测指标尚处于研究阶段，且对临床样本要求较高，检测、分析方法也较复杂，故有检测条件的中心可结合相关检测结果，对TMB指导肺癌免疫治疗进行综合解读。

**Table:** 

**本共识编撰指导专家：**	
**王洁**	**中国医学科学院肿瘤医院**
**周彩存**	**同济大学附属上海市肺科医院**
**周清华**	**四川大学华西医院**
**韩宝惠**	**上海交通大学附属胸科医院（上海市胸科医院）**
**宋勇**	**东部战区总医院**
**执笔专家：**	
**聂蔚**	**上海交通大学附属胸科医院（上海市胸科医院）**
**参与共识讨论的专家（按姓氏拼音排名）：**	
**操乐杰**	**中国科学技术大学附属第一医院（安徽省立医院）**
**储天晴**	**上海交通大学附属胸科医院（上海市胸科医院）**
**董晓荣**	**华中科技大学同济医学院附属协和医院**
**董宇超**	**上海长海医院**
**范云**	**中国科学院大学附属肿瘤医院**
**高蓓莉**	**上海交通大学医学院附属瑞金医院**
**谷伟**	**南京市第一医院（南京医科大学附属南京医院）**
**郭人花**	**江苏省人民医院（南京医科大学第一附属医院）**
**韩昱晨**	**上海交通大学附属胸科医院（上海市胸科医院）**
**何勇**	**陆军特色医学中心**
**胡晓桦**	**广西医科大学第一附属医院**
**胡毅**	**解放军总医院肿瘤医学部**
**黄建安**	**苏州大学附属第一医院**
**季枚**	**常州市第一人民医院（苏州大学附属第三医院）**
**金时**	**中国医学科学院肿瘤医院深圳医院**
**柯明耀**	**厦门医学院附属第二医院**
**李凯**	**天津医科大学肿瘤医院**
**李琳**	**北京医院**
**李伟峰**	**中国人民解放军南部战区总医院**
**李雯**	**浙江大学医学院附属第二医院**
**李晓燕**	**首都医科大学附属北京天坛医院**
**李媛**	**复旦大学附属肿瘤医院**
**林冬梅**	**北京大学肿瘤医院**
**林丽珠**	**广州中医药大学第一附属医院**
**刘安文**	**南昌大学第二附属医院**
**刘先领**	**中南大学湘雅二医院**
**吕冬青**	**温州医科大学附属台州医院**
**马学真**	**青岛市中心医院**
**茅国新**	**南通大学附属医院**
**聂立功**	**北京大学第一医院**
**潘宏铭**	**浙江大学医学院附属邵逸夫医院**
**沙丹**	**山东第一医科大学附属省立医院**
**史美祺**	**江苏省肿瘤医院**
**孙耕耘**	**安徽医科大学第一附属医院**
**田攀文**	**四川大学华西医院**
**王佳蕾**	**复旦大学附属肿瘤医院**
**王凯**	**浙江大学医学院附属第四医院**
**王立峰**	**南京大学医学院附属鼓楼医院**
**王启鸣**	**河南省肿瘤医院**
**王哲海**	**山东省肿瘤医院**
**吴宏成**	**宁波市医疗中心李惠利医院**
**杨秋安**	**山东大学齐鲁医院**
**尤长宣**	**南方医科大学南方医院**
**于起涛**	**广西医科大学附属肿瘤医院**
**岳东升**	**天津医科大学肿瘤医院**
**臧远胜**	**上海长征医院**
**张国俊**	**郑州大学第一附属医院**
**张力**	**中国医学科学院北京协和医院**
**张声**	**福建医科大学附属第一医院**
**张伟**	**南昌大学第一附属医院**
**张晓菊**	**河南省人民医院**
**张予辉**	**首都医科大学附属北京朝阳医院**
**赵军**	**北京大学肿瘤医院**
**钟华**	**上海交通大学附属胸科医院（上海市胸科医院）**
**周承志**	**广州医科大学附属第一医院**
**周建英**	**浙江大学医学院附属第一医院**
**朱波**	**陆军军医大学第二附属医院**
**庄武**	**福建省肿瘤医院（福建医科大学附属肿瘤医院）**
